# The role of PCOS in mental health and sexual function in women with obesity and a history of infertility

**DOI:** 10.1093/hropen/hoab038

**Published:** 2021-10-22

**Authors:** M D A Karsten, V Wekker, H Groen, R C Painter, B W J Mol, E T M Laan, T J Roseboom, A Hoek

**Affiliations:** 1 Department of Obstetrics and Gynaecology, University of Groningen, University Medical Centre Groningen, Groningen, The Netherlands; 2 Department of Obstetrics and Gynaecology, Amsterdam UMC, University of Amsterdam, Amsterdam, The Netherlands; 3 Amsterdam Reproduction and Development Research Institute, Amsterdam UMC, Amsterdam, The Netherlands; 4 Department of Clinical Epidemiology, Biostatistics and Bioinformatics, Amsterdam UMC, University of Amsterdam, Amsterdam, The Netherlands; 5 Amsterdam Public Health Research Institute, Amsterdam UMC, Amsterdam, The Netherlands; 6 Department of Epidemiology, University of Groningen, University Medical Centre Groningen, Groningen, The Netherlands; 7 Department of Obstetrics and Gynaecology, Monash Medical Centre, Monash University, Clayton, VIC, Australia; 8 Department of Sexology and Psychosomatic Gynaecology, Amsterdam UMC, University of Amsterdam, Amsterdam, The Netherlands

**Keywords:** polycystic ovary syndrome, obesity, quality of life, anxiety, depression, sexual dysfunction

## Abstract

**STUDY QUESTION:**

Do mental health and sexual function differ between women with or without polycystic ovary syndrome (PCOS) with comparable BMI and fertility characteristics?

**SUMMARY ANSWER:**

Women with PCOS have a poorer mental quality of life than women without PCOS, but there were no differences in symptoms of depression, anxiety, physical quality of life or sexual function.

**WHAT IS KNOWN ALREADY:**

Various studies suggest that women with PCOS have poorer mental health, such as higher symptoms of anxiety and depression with a lower quality of life, and have an impaired sexual function compared to women without PCOS. However, in most studies, BMI and infertility status differ between women with and without PCOS, which may hamper comparability.

**STUDY DESIGN, SIZE, DURATION:**

This study is a cross-sectional analysis of a 5-year follow-up of a randomized controlled trial (RCT) among women with obesity and a history of infertility.

**PARTICIPANTS/MATERIALS, SETTING, METHODS:**

Participants in this follow-up study of an RCT were women with obesity and infertility randomized to a lifestyle intervention followed by infertility treatment or prompt infertility treatment (control), stratified by ovulatory status and trial centre. In total, 173 (30.0%) women of the 577 women randomized in the initial trial participated in this follow-up study, with a mean follow-up of 5.5 years (range 3.7–7.0 years); of these women 73 had been diagnosed with PCOS and 100 did not have PCOS. Participants completed questionnaires on symptoms of anxiety and depression (Hospital Anxiety and Depression scale (HADS)), quality of life (36-item Short Form Health Survey (SF-36)) and sexual function (McCoy Female Sexuality Questionnaire (MFSQ)). We also compared quality of life subscale scores in women with and without PCOS and compared them to an age-matched Dutch reference population with average BMI. Effect sizes were calculated to assess the differences.

**MAIN RESULTS AND THE ROLE OF CHANCE:**

Symptoms of anxiety and depression, physical quality of life and sexual function did not differ significantly between obese women with and without PCOS. However, women with PCOS had a worse mental quality of life summary component score (−3.60 [95% CI −6.72 to −0.56]), mainly due to a lower score on the subscale ‘role limitations due to emotional problems’ (−12.41 [95% CI −22.78 to −2.28]), compared to women without PCOS. However, compared to an age-matched Dutch reference population, the obese infertile women with and without PCOS both scored lower on almost all physical and mental quality of life subscales.

**LIMITATIONS, REASONS FOR CAUTION:**

These are secondary analyses of the follow-up study of the RCT. No power analysis was performed for the outcomes included in this analysis and, as our study had a relatively small sample size, the null findings could be based on insufficient power to detect small differences between the groups. Our study population had a high mean BMI (average total group 34.5 [SD ± 5.1]); therefore, our results may only be generalizable to women with obesity.

**WIDER IMPLICATIONS OF THE FINDINGS:**

Our results indicate that PCOS status is associated with impaired mental quality of life. Anxiety and depression, physical quality of life and sexual function in obese infertile women with PCOS seem more related to the obesity than the PCOS status.

**STUDY FUNDING/COMPETING INTEREST(S):**

The initial study and follow-up were supported by grants from: ZonMw (50-50110-96-518), the Dutch Heart Foundation (2013T085) and the European Commission (633595). The Department of Obstetrics and Gynaecology of the UMCG received an unrestricted educational grant from Ferring pharmaceuticals BV, The Netherlands, outside the submitted work. A.H. reports consultancy for Ferring pharmaceuticals. B.W.J.M. is supported by an NHMRC Practitioner Fellowship (GNT1082548). B.W.J.M. reports consultancy for ObsEva, Merck Merck KGaA, iGenomix and Guerbet. All other authors declare no competing interests.

**TRIAL REGISTRATION NUMBER:**

The initial trial was registered on 16 November 2008 in the Dutch trial register; clinical trial registry number NTR1530.

WHAT DOES THIS MEAN FOR PATIENTS? Various studies suggest that women with polycystic ovary syndrome (PCOS) have poorer mental health, with more anxiety and depression, and lower quality of life. Studies also suggest that women with PCOS have more sexual problems, due to reduced levels of sexual arousal or lubrication, compared to women without PCOS. Previous studies looking into the effect of PCOS on mental health and sexuality have included women who varied in BMI and were either fertile or infertile. Since BMI and infertility have their own effect on mental health and sexuality, it is difficult to study the effects of PCOS alone in such studies. The advantage of our study is that we asked women with and without PCOS, with a similar BMI and history of infertility, to participate in our study. Women with PCOS experienced a poorer mental quality of life compared to the women without PCOS. Based on our findings, PCOS does not seem to affect sexuality, symptoms of depression or anxiety, or physical quality of life. We also compared all women who participated in our study, regardless of their PCOS status, to women of similar age from the general population, and found that the women who participated in our study reported more symptoms of anxiety and depression, poorer quality of life and more sexual problems. Therefore, obesity or infertility might have a bigger impact on mental health and sexuality than PCOS.

## Introduction

Polycystic ovary syndrome (PCOS) affects 5–20% of all women of reproductive age worldwide. It is a heterogeneous disorder characterized by three key features: anovulation, hyperandrogenism and the presence of polycystic ovaries. PCOS is a chronic disorder during the reproductive lifespan, commonly associated with acne, excessive growth of body hair (hirsutism), menstrual disturbance, infertility, obesity, insulin resistance, diabetes, dyslipidaemia, hypertension and metabolic syndrome, which is associated with long-term sequelae such as a higher risk of endometrial cancer, type II diabetes mellitus and cardiovascular disease later in life (Teede *et al.*, 2018).

Studies suggest that PCOS and its associated symptoms can have a negative effect on mental health, including higher rates of depressive symptoms and anxiety and a lower quality of life (Jones *et al.*, 2008; Veltman-Verhulst *et al.*, 2012; Cooney and Dokras, 2017; Teede *et al.*, 2018). Similar negative effects of PCOS symptoms can influence sexual function (Pastoor *et al.*, 2018). Clinical features of PCOS, such as acne, hirsutism, obesity and infertility can induce emotional distress (Conaglen and Conaglen, 2003; Chachamovich *et al.*, 2010), thereby reduce mental health and sexual function (Luppino *et al.*, 2010; Kolotkin *et al.*, 2012; Lim *et al.*, 2013; Pastoor *et al.*, 2018).

Confounding by obesity and infertility are major limitations of previous studies investigating anxiety and depression, quality of life and sexual function in women with and without PCOS, since in many studies the PCOS groups have had a higher BMI and higher rates of infertility than controls (Veltman-Verhulst *et al.*, 2012; Teede *et al.*, 2018; Pastoor *et al.*, 2018).

The aim of the current study was therefore to investigate whether mental health and sexual function differ between women with or without PCOS with a comparable BMI and fertility characteristics. We therefore compared symptoms of anxiety and depression, quality of life and sexual function in women with obesity and a history of infertility with and without PCOS.

## Materials and methods

The current study is a cross-sectional analysis of data from a follow-up study after a multicentre randomized controlled trial (RCT) in the Netherlands. The study protocols of the initial RCT and the current follow-up study have been published previously (Mutsaerts *et al.*, 2010; van de Beek *et al.*, 2018). The initial RCT recruited outpatients of infertility clinics between June 2009 and June 2012. A total of 577 women aged 18–39 years with infertility and a BMI ≥29 kg/m^2^ were randomly (1:1) allocated to the intervention or control group.

Randomization for the initial RCT was performed by the Amsterdam University Medical Center with an online programme, was stratified for anovulatory/ovulatory status and trial centre, and could not be blinded due to the nature of the treatment allocation (Mutsaerts *et al.*, 2010). After randomization, the intervention group received a lifestyle intervention, which was followed by infertility treatment. The control group received immediate infertility treatment after randomization. Infertility treatment was performed according to the Dutch infertility guidelines, irrespective of their BMI (Dutch Society of Obstetrics and Gynaecology (NVOG), 2015).

Women assigned to the intervention group were offered a 6-month preconception lifestyle intervention that consisted of an energy restricted diet to reduce caloric intake with 600 kcal (not less than 1200 calories), encouragement to increase in physical activity up to 10 000 steps a day, 30 min of moderate to vigorous exercise twice to three times a week, and individualized behavioural counselling. The main goal was a weight reduction of at least 5% of their initial bodyweight, or a reduction in BMI below 29 kg/m^2^ within the intervention period of 6 months.

Five years (range 3.7–7.0 years) after randomization in the preconception lifestyle intervention trial (LIFEstyle study) (Mutsaerts *et al.*, 2010, 2016), women participated in the current follow-up study (WOMB project) (van de Beek *et al.*, 2018). All women who participated in the initial trial were approached to participate in the follow-up study. The follow-up study included a wide range of physical, mental, dietary and physical activity health measures. The current evaluation includes questionnaires that were filled out by women at home (on paper or online) without the presence of a researcher (van de Beek *et al.*, 2018).

### Participants

Women with infertility and a BMI ≥29 kg/m^2^ participated in this follow-up study. PCOS was diagnosed by clinicians at entry within the initial trial, based on the Rotterdam 2003 criteria (Fauser, 2004). The women in the control group of this study consisted of women with obesity and infertility without PCOS (ovulatory and anovulatory non-PCOS women (WHO class I and II)). Infertility was defined as chronic anovulation according to WHO class I/II (The ESHRE Capri Workshop Group, 1995) or PCOS (Fauser, 2004) or unsuccessful conception after unprotected intercourse for at least 12 months (Dutch Society of Obstetrics and Gynaecology (NVOG), 2015). A detailed description of the initial trial and main results (Mutsaerts *et al.*, 2010, 2016) and follow-up trial design (van de Beek *et al.*, 2018) have been published previously.

### Mental health

#### Anxiety and depression

Anxiety and depression symptoms were assessed with the Dutch translation of the 14-item Hospital Anxiety and Depression Scale (HADS) (Zigmond and Snaith, 1983; Spinhoven *et al.*, 1997). The HADS is a self-report rating scale that consists of two 7-item subscales measuring summarized anxiety and depression. For each item, a 4-point response scale is used: 0 (absence of symptoms) to 3 (maximum symptomatology), with a theoretical range from 0 to 21 for each subscale. The total score is calculated as sum of the 14 items. Higher scores indicate a higher level of anxiety or depression. Anxiety or depression is considered to be present at a HADS score of 8 or above. The percentage of women exceeding the cut-off score (≥8) for both anxiety and depression was calculated (Hinz and Brähler, 2011). Scores from 8–10 represent mild, 11–14 represent moderate and 15–21 represent severe anxiety or depression (Zigmond and Snaith, 1983). The HADS has demonstrated good reliability for both depression (*α* = 0.82) and anxiety (*α* = 0.83) in a large validation study (Bjelland *et al.*, 2002).

#### Quality of life

Quality of life was assessed with the Dutch translated version of the 36-Item Short Form Health Survey (SF-36) (Wendel-Vos *et al.*, 2003). The SF-36 is a 36-item questionnaire with eight multi-items scales (Aaronson *et al.*, 1998). A Physical Component Summary (PCS) and a Mental Component Summary (MCS) were calculated, in which higher scores indicate a better quality of life. The following subscales were calculated from the PCS: ‘physical functioning’ (10 items), ‘role limitations due to physical health’ (4 items), ‘bodily pain’ (2 items), ‘general health’ (5 items). From the MCS, the subscales ‘social functioning’ (2 items), ‘role limitations due to emotional problems’ (3 items), ‘mental health/emotional well-being’ (5 items) and ‘vitality’ (4 items) were calculated. Each scale is transformed linearly into a 0–100 scale on the assumption that each question carries equal weight. The Dutch version of the SF-36 is widely used and demonstrates good reliability; Cronbach’s *α* = 0.71 to 0.92 (Vanderzee *et al.*, 1996).

To compare quality of life in women with and without PCOS to an age-matched Dutch reference population (Aaronson *et al.*, 1998), we calculated mean differences and converted the eight subscales of the SF-36 to standard scores. The mean differences were calculated by the difference between mean SF-36 scores of both women with and without PCOS and the mean score of the age-matched reference population. Mean differences <0 indicate that quality of life is worse than that of the age-matched Dutch reference population. Effect sizes were calculated by dividing the mean difference between the patients’ SF-36 score and the mean score of the matched reference population by the SDs of the reference population (mean difference/SD). Effect sizes of 0.20, 0.50, 0.80 and 1.20 are considered to indicate a small, moderate, large or very large deviation from the reference population (Cohen, 1988; Sawilowsky, 2009).

### Sexual function

Sexual function was assessed using the validated Dutch version of the McCoy Female Sexuality Questionnaire (MFSQ) (McCoy, 2000). The MFSQ is a 19-item questionnaire of which 18 items are answered on a 7-point Likert scale and one item evaluates the intercourse frequency over the past 4 weeks. The MFSQ consists of five subdomains: sexual interest (4 items), vaginal lubrication (3 items), orgasm (4 items), satisfaction with frequency of sexual activity (3 items) and sex partner (3 items on satisfaction with partner as friend and lover and erectile problems of partner). Intercourse frequency is converted to a 7-point Likert scale on a percentage wise basis, as part of the satisfaction domain. All 19 items could be completed when a woman engaged in vaginal intercourse over the past 4 weeks. Items 1 to 11 could be answered by women who were both sexually inactive and sexually active (McCoy, 2000). To calculate the domain-specific sum scores, the ‘non-applicable’ MFSQ-items were replaced with the mean of at least two other items within that subdomain. The total MFSQ score was calculated by the sum of all individual items, with a theoretical range from 19 to 133 points in total. In accordance with the questionnaire instructions, no total MFSQ scores were calculated for women who did not engage in vaginal intercourse during the last four weeks. A higher MFSQ total score indicates better sexual function. The MFSQ is able to discriminate between women with and without sexual dysfunction (Giraldi *et al.*, 2011) and has a good test–retest reliability (Pearson *r* = 0.71–0.95).

### Statistical analysis

Demographic characteristics are reported as means and SD for continuous variables. For categorical variables, frequencies are reported as number of participants (n) and percentage. Continuous variables were analysed with the independent sample *T*-test or Mann–Whitney *U* test and categorical and binary outcomes were analysed with the Chi-square, Fisher’s exact test or Fisher–Freeman–Halton exact test.

The Kolmogorov–Smirnov test was used to test whether the outcomes were normally distributed. In case the normality assumption was violated, results are presented as mean difference with bias-corrected and accelerated 95% CI’s, based on 5000 bootstrap re-samples (Diciccio and Efron, 1996). CIs not including zero were considered to indicate statistical significance. Linear regression was performed to examine associations between PCOS status (binary) and continuous MFSQ outcomes. As the initial intervention aimed to reduce weight, it could influence sexual function. Furthermore, whether or not women were still attempting to conceive was also thought to influence sexual function. Being in the intervention or control group (binary) and attempting to conceive (binary) were therefore added as covariates to the adjusted model for the MFSQ outcomes, as they indeed changed the estimates. Results are presented as mean difference and the corresponding 95% CI. Having had a child was also considered as a covariate. Although it did not appear to change the estimates for any of the outcomes, it could also be a mediator, but this was not explored further.

A sensitivity analysis was performed comparing PCOS women to ovulatory women as controls, excluding 16 women classified as WHO class II anovulation but without PCOS from this control group, as different WHO II anovulation status may influence mental health and sexual function outcomes. A second sensitivity analysis was performed to rule out the influence of Selective Serotonin Reuptake Inhibitors (SSRIs) and Serotonin Noradrenalin Reuptake Inhibitors (SNRIs) (n = 8) on the MFSQ, HADS and SF-36 outcomes. We excluded women who used these medications from this analysis, as these medications are known to influence mental health and sexual function. A third sensitivity analysis was performed by calculating an adapted MFSQ total score from items 1 to 11. These items could be answered by all women, irrespective of whether they were sexually active or not. As a result, we could include those women who reported that they did not engage in vaginal intercourse in the past 4 weeks (n = 30) and were excluded from the main analyses in accordance with the correct use of the MFSQ questionnaire. We calculated MFSQ total scores and domain scores for these comparisons.

*P*-values <0.05 were considered as statistically significant. Statistical analyses were performed using SPSS version 24 for Windows (IBM Corp., Armonk, NY, USA).

### Ethical approval

The LIFEstyle RCT as well as the WOMB project follow-up study were approved by the institutional medical ethics review committee of the University Medical Center Groningen (METc code: 2008/284). The LIFEstyle study was registered on 16 November 2008 in the Dutch trial register (clinical trial registry number: NTR1530). Written informed consent was given by all participants at both the beginning of the LIFEstyle RCT and the WOMB project follow-up. Both the initial study and follow-up were conducted according to the principles of the Declaration of Helsinki.

## Results

### Flow of participants

**Figure 1.**
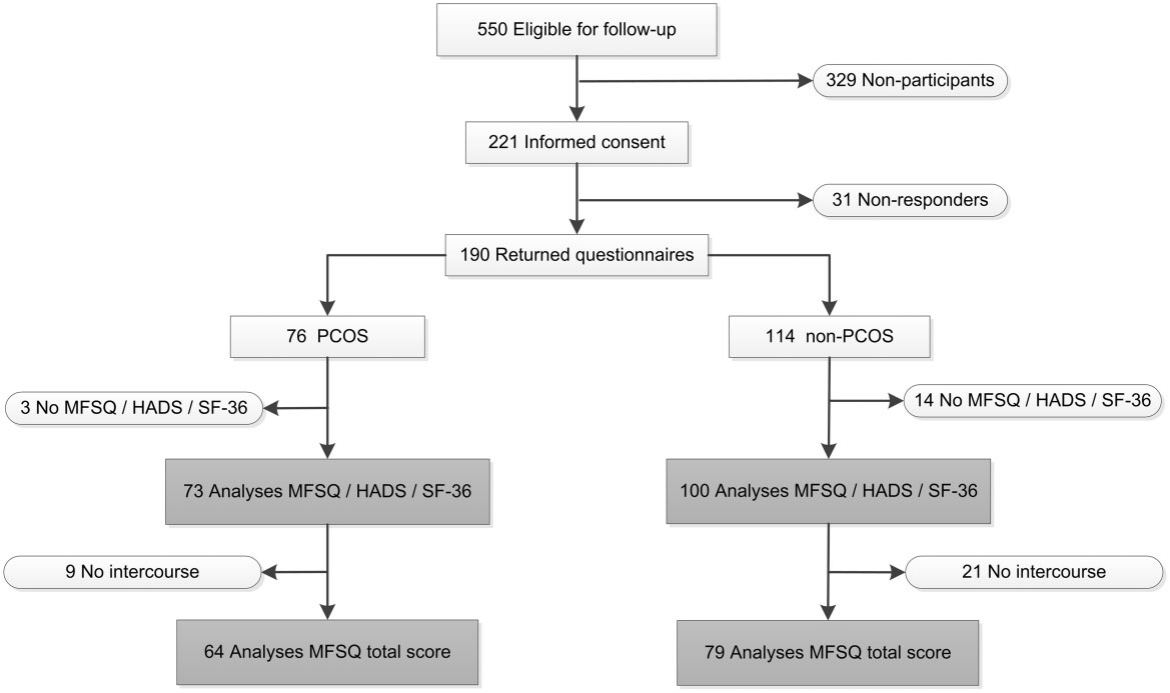
**Flow of participants.** HADS, Hospital Anxiety and Depression Scale; MFSQ, McCoy Female Sexuality Questionnaire; PCOS, Polycystic ovary syndrome; SF-36, 36-Item Short Form Health Survey.

In total, 190 women of the 577 women returned questionnaires (32.9%) during this follow-up study with a mean follow-up of 5.5 years (range 3.7–7.0 years). Women who participated in our follow-up study had a better mental quality of life, were more often of Caucasian origin and had been trying to conceive for less time (see Supplementary Table SI for a further comparison of baseline characteristics of participants and non-participants). Of the 190 women who responded to the follow-up questionnaire, 17 women did not return the complete set of questionnaires for unspecified reasons. Thus, 173 women were included in the analysis. Of these, 30 women did not have intercourse in the past 4 weeks and were therefore excluded from the analyses as we could not compute an MFSQ total score (see Fig. 1).

### Characteristics of participants

Table I displays the characteristics of participants in this follow-up. Overall, 73 (42.2%) women with PCOS and 100 (57.8%) ovulatory and anovulatory women without PCOS were included. There were no differences in characteristics between the PCOS and non-PCOS group. The average age was 34.7 years with an average BMI of 34.5 kg/m^2^.

**Table I:** Characteristics of participants during follow-up.

Characteristics	Total group	PCOS^a^	Non-PCOS^b^	P-value^c^
	(n = 173)	(n = 73)	(n = 100)	
Age, years (mean; SD)	34.7 (3.9)	34.1 (4.1)	35.1 (3.7)	0.12
Caucasian (yes; %; n)	95.4 (165)	95.9 (70)	95.4 (95)	0.08
BMI (kg/m^2^; mean; SD)	34.5 (5.1)	34.6 (5.3)	34.4 (4.9)	0.82
Waist circumference (cm; mean; SD)	107.7 (13.5)	108.2 (12.8)	107.4 (14.0)	0.73
Hip circumference (cm; mean; SD)	120.2 (13.1)	121.1 (13.5)	119.6 (12.9)	0.48
Education level (%; n)				0.56
No education/Primary school (age 4–12 years)	2.9 (5)	4.1 (3)	2.0 (2)	
Secondary education	19.1 (33)	15.1 (11)	22.0 (22)	
Intermediate Vocational Education	53.2 (92)	53.4 (39)	53.0 (53)	
Higher Vocational Education or University	24.9 (43)	27.4 (20)	23.0 (23)	
Current smoker (yes; %; n)	16.8 (29)	15.1 (11)	18.0 (18)	0.24
Infertility diagnosis (%; n)				NA
Anovulatory^d^	51.4 (89)	100.0 (73)	16.0 (16)	
Unexplained	27.7 (48)	0.0 (0)	48.0 (48)	
Male factor	21.4 (37)	8.2 (6)	31.0 (31)	
Tubal factor	3.5 (6)	0.0 (0)	6.0 (6)	
Has a partner (yes; %; n)	97.1 (168)	97.3 (71)	97.0 (97)	0.65
Attempting to conceive (yes; %; n)^e^	26.1 (37)	19.2 (14)	23.0 (23)	0.74
Has a child (yes; %; n)	80.9 (140)	84.9 (62)	78.0 (78)	0.25

PCOS, polycystic ovary syndrome; NA, not applicable.

^a^ Diagnosed by Rotterdam 2003 criteria (Fauser, 2004).

^b^ Non-PCOS: ovulatory and anovulatory non-PCO women (WHO class I and II).

^c^ Differences between women with PCOS and controls were analysed with the independent sample *t*-test for continuous variables or the Chi-square test or Fisher’s exact test for categorical variables.

^d^ Ovulatory status was assessed at randomization.

^e^ Attempting to conceive was examined by a single question ‘Are you trying to become pregnant right now?’.

### PCOS and mental health

Symptoms of anxiety and depression measured by the HADS did not differ between women diagnosed with PCOS and women without PCOS (Table II). Using the cut-off (score ≥ 8) for the presence of anxiety- and depressive disorder, 35 women (47.9%) with PCOS and 47 women (47.0%) without PCOS scored above the cut-off for anxiety. Moreover, 35 women (47.9%) with PCOS and 37 women (37.0%) without PCOS scored above the cut-off for depression. There were no significant differences between the groups in the number of women with anxiety and depression scores above the cut-off (results not shown).

**Table II:** Anxiety and depression in women with and without PCOS.

Anxiety and depression outcomes (HADS)	**PCOS** ^a^	**Non-PCOS** ^b^	**Mean difference** ^c^	**95% CI** ^d^
	(n = 73)	(n = 100)		
Symptoms of anxiety (score; mean; SD)	8.3 (3.9)	8.1 (3.4)	0.26	−0.85 to 1.37
Symptoms of depression (score; mean; SD)	8.2 (3.7)	7.4 (3.2)	0.77	−0.28 to 1.87
HADS total score (score; mean; SD)	16.5 (7.0)	15.4 (6.1)	1.03	−0.92 to 3.02

HADS, Hospital Anxiety and Depression scale; PCOS, polycystic ovary syndrome.

^a^ Diagnosed by Rotterdam 2003 criteria (Fauser, 2004).

^b^ Non-PCOS: ovulatory and anovulatory non-PCO women.

^c^ The mean difference between the PCOS and non-PCOS group was assessed with the independent sample *t*-test.

^d^ Bias corrected and accelerated 95% CIs based on 5000 bootstrap re-samples; CI not containing zero indicate statistical significance.

**Figure 2.**
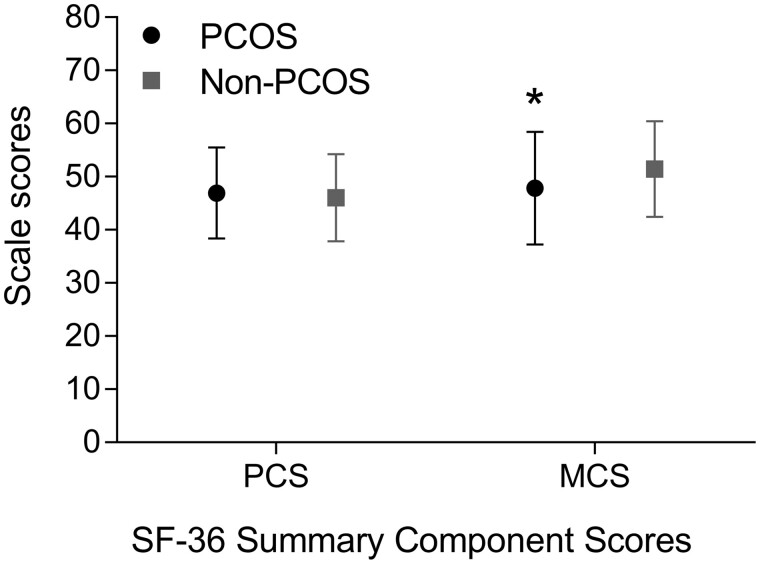
**Physical and Mental Component Summary domains measured with the SF-36 in women with PCOS and without PCOS**. Data are presented as means, error bars indicate SD. *Significant difference in MCS between PCOS and non-PCOS group: −3.60 points [95% CI −6.72 to −0.56]; *P* = 0.02 analysed with the independent sample *t*-test. Bias corrected and accelerated 95% CIs based on 5000 bootstrap re-samples; confidence intervals not containing zero indicate statistical significance. Mean difference PCS between women with PCOS and without PCOS: 0.89 points [95% CI −1.72 to 3.40]; *P* = 0.50. MCS, Mental Component Summary; PCOS, polycystic ovary syndrome; PCS, Physical Component Summary; SF-36, 36-Item Short Form Health Survey.

**Figure 3.**
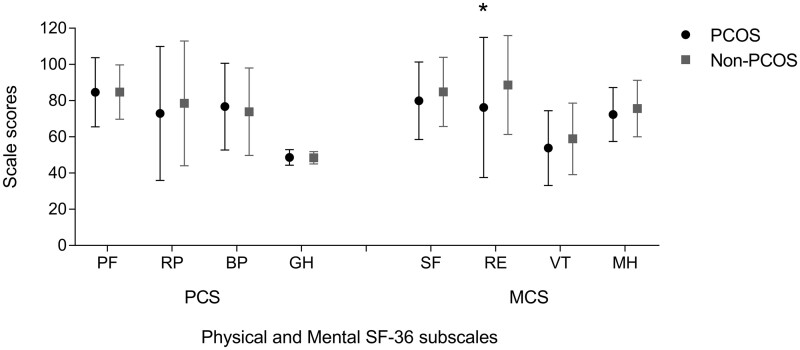
**SF-36 subscale scores in women with and without PCOS**. Subdomains of the SF-36 PCS (Physical Component Summary): PF, physical functioning; RP, role limitations due to physical health; BP, bodily pain; GH, general health. Subdomains of the SF-36 MCS (Mental Component Summary): SF, social functioning; RE, role limitations due to physical health; VT, vitality; MH, mental health/emotional well-being. All data are presented as mean and SD. *Significant difference in RE between PCOS and non-PCOS group (mean difference: −12.41 points [95% CI −22.78 to −2.28]; *P* < 0.01) analysed with the independent sample *t*-test. Bias corrected and accelerated 95% CIs based on 5000 bootstrap re-samples; confidence intervals not containing zero indicate statistical significance. Mean difference between women with and without PCOS and corresponding 95% CI in other PCS subscales: PF −0.09 [−5.41 to 5.21]; RP −5.56 [−16.54 to 5.20]; BP 2.84 [−4.53 to 10.08]; GH 0.18 [−0.97 to 1.36]. Mean difference between women with and without PCOS and corresponding 95% CI in other MCS subscales: SF −4.91 [−10.90 to 1.24]; VT −5.06 [−11.19 to 1.00]; MH −3.35 [−7.98 to 1.16]. PCOS, polycystic ovary syndrome; SF-36, 36-Item Short Form Health Survey.

With regard to quality of life measured by the SF-36, the summary- and separate subscales for physical quality of life were not different in women with and without PCOS. However, women with PCOS had a significantly lower MCS score compared to women without PCOS (−3.60 points [95% CI −6.72 to −0.56]; Fig. 2). Furthermore, the subscale ‘role limitations due to emotional problems’ within the mental quality of life component summary was significantly lower in women with PCOS (−12.41 points [95% CI −22.78 to −2.28]) (Fig. 3).

### Quality of life compared to the reference population

Relative to an age-matched Dutch reference population wherein data were collected between 1992 and 1996 (Aaronson *et al.*, 1998), both our groups of women with and without PCOS scored lower on all physical quality of life subscales, indicating a worse quality of life (Table IV). Effect sizes showed small to very large deviations in physical quality of life from the reference population in women with and without PCOS and obesity.

**Table IV:** Quality of life in obese infertile women with and without PCOS compared to an age-matched Dutch reference population.

Quality of life outcomes (SF-36)	**Age-matched Dutch reference population** ^¥^	**PCOS** ^a^ (n = 73)	Mean difference^∑^	Effect size*	**Non-PCOS** ^b^ (n = 100)	Mean difference^∑^	Effect size*
			**(PCOS vs.** reference)			(non-PCOS vs. reference)	
*Physical Component Summary, score, mean (SD)*	NA	46.9 (8.5)	NA	NA	46.0 (8.2)	NA	NA
Physical functioning, score, mean (SD)	93.1 (11.8)	84.6 (19.1)	−8.5	0.72	84.8 (15.0)	−8.3	0.70
Role limitations due to physical health, score, mean (SD)	86.4 (27.6)	73.0 (37.0)	−13.4	0.49	78.5 (34.5)	−7.9	0.29
Bodily pain, score, mean (SD)	80.9 (19.4)	76.7 (24.0)	−4.2	0.22	73.9 (24.1)	−7.0	0.36
General health, score, mean (SD)	78.2 (17.3)	48.6 (4.3)	−29.6	1.71	48.5 (3.4)	−29.7	1.72
*Mental Component Summary, score, mean (SD)*	NA	47.8 (10.6)	NA	NA	51.4 (9.0)	NA	NA
Social functioning domain, score, mean (SD)	87.8 (19.1)	80.0 (21.4)	−7.8	0.41	84.9 (19.1)	−2.9	0.15
Role limitations due to emotional problems, score, mean (SD)	85.4 (30.0)	76.3 (38.7)	−9.1	0.30	88.7 (27.3)	3.3	0.11
Vitality domain, score, mean (SD)	70.7 (16.4)	53.8 (20.7)	−16.9	1.03	58.9 (19.7)	−11.8	0.72
Mental health/emotional well-being, mean (SD)	78.7 (15.2)	72.3 (14.9)	−6.4	0.42	75.7 (15.6)	−3.0	0.20

PCOS, polycystic ovary syndrome; SF-36, 36-Item Short Form Health Survey; NA, not applicable.

^¥^ Dutch reference population of females and males <40 years (Aaronson *et al.*, 1998).

^∑^ Mean differences <0 indicates that quality of life is worse than that of the age-matched Dutch reference population. Mean differences were calculated by the difference between SF-36 scores and the mean score of the reference population.

^a^ Diagnosed by Rotterdam 2003 criteria (Fauser, 2004).

^b^ Non-PCOS: ovulatory and anovulatory non-PCO women.

* Effect sizes indicate the difference in SF-36 scores between PCOS and non-PCOS groups and the reference population, expressed in SD’s (difference/SD). Effect sizes of 0.20, 0.50, 0.80 and 1.20 are considered to indicate a small-, moderate-, large- or very large deviation from the reference population (Cohen, 1988; Sawilowsky, 2009).

Furthermore, lower scores with small to large effect sizes were seen in all mental quality of life subscales in women with PCOS, with the highest large effect sizes in the subscales ‘vitality’ and ‘role limitations due to emotional problems’.

In women without PCOS and obesity, all mental quality of life subscales were lower than the reference population, except the subscale ‘role limitations due to emotional problems’ in which women with obesity but without PCOS scored higher than the reference population.

### PCOS and sexual function

Within all sexual function domain scores and the MFSQ total score, no difference in sexual function nor in sexual intercourse frequency was seen between women diagnosed with PCOS and women without PCOS. Adjusting for the randomization group and ‘attempting to conceive’ did not change these estimates (Table III).

**Table III:** Sexual function and intercourse frequency in women with and without PCOS.

					Unadjusted		**Adjusted** ^a^	
Sexual function outcomes (MFSQ)	n	**PCOS** ^b^	n	**Non-PCOS** ^c^	**Mean difference** ^d^	**95% CI** ^e^	**Mean difference** ^d^	**95% CI** ^e^
Sexual interest (score; mean; SD)^*^	73	26.4 (6.6)	100	25.4 (7.4)	0.95	−1.17 to 3.07	0.29	−1.93 to 2.60
Sexual satisfaction (score; mean; SD)^*^	70	11.2 (2.5)	97	10.9 (3.0)	0.38	−0.45 to 1.21	0.25	−0.59 to 1.09
Vaginal lubrication (score; mean; SD)	64	16.4 (3.0)	79	15.6 (3.5)	0.79	−0.26 to 1.87	0.97	−0.19 to 2.12
Orgasm, score (score; mean; SD)	64	20.4 (4.8)	79	19.9 (5.4)	0.45	−1.21 to 2.10	0.65	−1.16 to 2.46
Sex partner, score (score; mean; SD)^*^	70	18.9 (2.1)	95	18.7 (2.7)	0.14	−0.60 to 0.87	0.02	−0.83 to 0.85
Total MFSQ (score; mean; SD)^¥^	64	94.4 (13.5)	79	93.3 (14.1)	1.09	−3.60 to 5.67	0.57	−4.29 to 5.47
Intercourse frequency (number per 4 weeks; mean; SD)	64	6.0 (5.8)	79	5.6 (4.4)	0.40	−1.25 to 2.22	−0.34	−2.04 to 1.48

MFSQ, McCoy Female Sexuality Questionnaire; PCOS, polycystic ovary syndrome.

^a^ Adjusted for randomization group within the initial RCT and for attempting to conceive.

^b^ Diagnosed by Rotterdam 2003 criteria (Fauser, 2004).

^c^ Non-PCOS: ovulatory and anovulatory non-PCO women (WHO class I and II).

^d^ The mean difference between the PCOS and non-PCOS group was assessed with linear regression.

^e^ Bias corrected and accelerated 95% CIs based on 5000 bootstrap re-samples; CI not containing zero indicate statistical significance.

^¥^ For women who have had sexual intercourse only.

* Analyses in women who have had sexual intercourse only show the same results (PCOS n = 64; non-PCOS n = 79).

### Sensitivity analyses

Sensitivity analyses comparing PCOS women to ovulatory controls were performed, by excluding 16 anovulatory women who did not meet the WHO class II criteria for PCO from the control group; this did not change the results for anxiety and depression (Supplementary Table SII), quality of life (Supplementary Table SIII), nor sexual function and sexual intercourse frequency (Supplementary Table SIV). Furthermore, a second sensitivity analysis was performed excluding women who used SSRI or SNRI antidepressant medication (n = 8); this also did not change the results for anxiety and depression, quality of life, nor sexual function and intercourse frequency. After exclusion of these women, both the MCS (mean difference −3.60 points in women with PCOS [95% CI −6.54 to −0.56]) and the mental quality of life subscale ‘role limitations due to emotional problems’ (mean difference −13.78 points in women with PCOS [95% CI −24.34 to −3.78]) remained significantly lower in women with PCOS compared to women without PCOS. The third sensitivity analysis, involving the adapted MFSQ score from items 1 to 11, did not substantially change the results of our main analysis regarding the adapted total MFSQ score and the adapted subdomain scores (results not shown).

## Discussion

Amongst women with obesity and a history of infertility, we found no difference in anxiety and depression, physical quality of life and sexual function in women with or without PCOS. However, obese women with a history of infertility and PCOS had an impaired mental quality of life, particularly in the subscale ‘role limitations due to emotional problems’ compared to obese women with a history of infertility but no PCOS. Our results indicate that anxiety and depression, physical quality of life and sexual function in women appears not to be associated with PCOS status. However, PCOS status seems to be associated with impaired mental quality of life.

In our study, we did not find an effect of a PCOS diagnosis on depressive symptoms nor on symptoms of anxiety in women without PCOS with a comparable high BMI and history of infertility. However, when comparing the prevalence of anxiety and depression (cut-off score ≥ 8) to an age- and sex-matched representative German population in which data were collected in 1998 (subsample 1) and 2009 (subsample 2) (Hinz and Brähler, 2011), 47.9% of women with PCOS and 47.0% of women without PCOS in our population meet the criteria for anxiety disorder compared with 19.8% in the reference population. Furthermore, 47.9% of women with PCOS and 47.0% of women without PCOS meet the criteria for depression compared with 12.8% in the reference population. This indicates that symptoms of anxiety and depression in this group seem to be more related to obesity and a history of infertility than to PCOS status. This is supported by the meta-analysis of Veltman-Verhulst *et al.* (2012), indicating that BMI, and also the degree of obesity, explains part of the effect on anxiety and depression scores. In a subgroup analysis of eight studies that had matched for BMI, these authors found lower standardized mean differences for depression than in studies in which BMI was higher in women with PCOS compared to controls (*P* = 0.0004) (Veltman-Verhulst *et al.*, 2012). A comparison of BMI categories showed that women with a BMI >30 had the highest depression scores (*P* = 0.001) compared to women in lower BMI categories (Veltman-Verhulst *et al.*, 2012). Although most women in our group (80%) gave birth during or after the trial, the desired family size may not have been met, which may have induced symptoms of anxiety and depression (Verhaak *et al.*, 2007).

With respect to quality of life among women with PCOS, our findings are in line with the only other study that addressed quality of life among women with and without PCOS with a similar high BMI (Álvarez-Blasco *et al.*, 2010). Álvarez-Blasco *et al.* (2010) did not find differences in physical quality of life between obese women with and without PCOS, but a similar negative effect of PCOS was found on mental quality of life in the subscale ‘role limitations due to emotional problems’. Role limitations due to emotional problems hamper women to optimally function in terms of work, social interactions, self-care and mobility and may thus importantly affect their wellbeing even while physical functioning itself was not impaired.

Compared to the age-matched Dutch reference population (Aaronson *et al.*, 1998), our group of women with PCOS had a lower mental quality of life on all subscales. The second lowest mental quality of life score and the second largest effect size was seen in women with PCOS and obesity on the subscale ‘role limitations due to emotional problems’ (Table IV). Women without PCOS in our study also scored lower on all mental quality of life subscales. However, on the subscale ‘role limitations due to emotional problems’ women with obesity but without PCOS scored higher than the reference population. This indicates that a high BMI and infertility seem to be important factors in impairing physical, as well as mental, quality of life in both women with and without PCOS (Fontaine and Barofsky, 2001; Sarwer *et al.*, 2012), and that PCOS is an additional factor in lowering mental quality of life. The mental quality of life subscale ‘role limitations due to emotional problems’ maps the impact of emotional problems on daily activities, such as spending less time on work, accomplishing less, and working less carefully than usual (for questions in this subscale see, Supplementary Table SV). It might be that in women with PCOS, the additional hormonal imbalance leading to anovulation as the reason for infertility leads to a higher experienced burden on daily activities, which is independent of their obesity.

No differences in sexual function or intercourse frequency were found in our study in obese women with and without PCOS. A recent review and meta-analysis showed small but significantly lower sexual function in women with PCOS compared to controls (Pastoor *et al.*, 2018). However, of the five studies in this meta-analysis that had controlled for BMI, mean BMI was considerably lower (mean BMI’s varying from 21 to 27 kg/m^2^) than in our study group. Findings of these five individual studies suggest that BMI rather than PCOS is associated with lower sexual function scores, with increasing degrees of obesity being associated with worse sexual function (Drosdzol *et al.*, 2007; Ercan *et al.*, 2013; Kowalczyk *et al.*, 2015; Lara *et al.*, 2015; Noroozzadeh *et al.*, 2017). There are little reference data for the version of the MFSQ questionnaire we used; the only study with a healthy control group suggests that our women with and without PCOS appear to have an impaired sexual function (Lundin *et al.*, 2018) (data collection between 2013 and 2015). Therefore, lower sexual function in these studies of obese women with PCOS could be due to obesity rather than the PCOS status itself.

A strength of our analysis is the comparable BMI and history of infertility of our groups. It also means, however, that our findings may only be generalizable to women with obesity and a history of infertility. Furthermore, we performed adjusted analyses and sensitivity analyses to control for potential confounding.

The limitations of this study deserve comment. PCOS was clinically diagnosed at randomization of the initial trial and was not assessed again during follow-up 5 years later. However, PCOS is assumed to be a lifelong condition (Teede *et al.*, 2010; Puurunen *et al.*, 2011). Furthermore, the cross-sectional analysis in this study did not allow us to assess causality. No power analysis was performed for the outcomes included in this analysis, and as our study had a relatively small sample size, therefore the null findings could be based on insufficient power to detect small differences between the groups. Women’s perception of their role in the aetiology of their infertility may have affected how they rated their quality of life. In women with PCOS, infertility is mainly due to anovulation, and this might have produced a sense of guilt in these women, maybe more than in women with other reasons for infertility, such as male factor infertility, and this could affect their mental quality of life. Those who declined to participate in the study showed lower mental health than those who did participate, therefore distress might therefore have been underestimated in our study sample. However, we found no further evidence that selective participation biased our results, as some adjustments for these different baseline characteristics (Supplementary Table SI) did not influence our findings.

To conclude, anxiety and depression, physical quality of life and sexual function in obese women with PCOS and a history of infertility seem more related to being obese or infertile than with PCOS status. However, mental quality of life is associated with PCOS status in these women, as women with PCOS (regardless of BMI) feel limited in their roles in life, which hampers their wellbeing. We cannot rule out that PCOS as a diagnosis and main factor of infertility in a couple, gives an additional burden to the mental quality of life in these women. Clinicians therefore may want to pay attention to the psychosocial dimension of PCOS. Future studies should validate our findings in a group of women with and without PCOS with matched BMIs and infertility histories.

## Supplementary data

Supplementary data are available at *Human Reproduction Open* online.

## Data availability

The data underlying this article will be shared on reasonable request to the corresponding author.

## Supplementary Material

hoab038_Supplementary_Data
