# Supplementary material for: The role of PCOS in mental health and sexual function in women with obesity and a history of infertility
Source: Hum Reprod Open. 2021 Oct 22;2021(4):hoab038. doi: 10.1093/hropen/hoab038 (PMC8643501; doi:10.1093/hropen/hoab038)
Supplement: hoab038_Supplementary_Data [file hoab038_supplementary_data.docx]

**Appendices**

| **Supplementary Table SI.** Comparison of baseline characteristics of participants and non-participants | | | | | | |
| --- | --- | --- | --- | --- | --- | --- |
| **Variables** | | **n** | **Non-participants** | **n** | **Participants** | **P-value ^a^** |
| Age (years; mean; SD) | | 397 | 29.7 (4.7) | 177 | 30.0 (4.2) | 0.45 |
| Weight (kg; mean; SD) | | 395 | 102.8 (13.3) | 177 | 104.2 (12.3) | 0.26 |
| BMI (kg/m2; mean; SD) | | 395 | 36.1 (3.5) | 177 | 36.0 (3.2) | 0.67 |
| Caucasian (n; %) | | 397 | 333 (83.9) | 177 | 169 (95.5) | <0.01 |
| Education (n; %) | | 377 |  | 172 |  | 0.07 |
|  | Primary school, age 4-12 year |  | 23 (6.1) |  | 4 (2.3) |  |
|  | Secondary education |  | 96 (25.5) |  | 35 (20.3) |  |
|  | Intermediate vocational education |  | 171 (45.4) |  | 95 (55.2) |  |
|  | Advanced vocational education or university |  | 87 (23.1) |  | 38 (22.1) |  |
| Current smoker (n; %) | | 394 | 99 (25.1) | 175 | 37 (21.1) | 0.30 |
| Nulliparous (n; %) | | 396 | 308 (77.8) | 177 | 133 (75.1) | 0.49 |
| Duration of infertility (median; IQR) | | 394 | 22.0 (14.0 – 36.0) | 177 | 19.0 (13.0 – 30.0) | 0.03 |
| Polycystic Ovary Syndrome ^b^ (n; %) | | 395 | 128 (32.4) | 177 | 73 (41.2) | 0.04 |
| Physical Quality of Life (median; IQR) | | 312 | 52.8 (47.8 – 55.9) | 151 | 52.1 (46.4 – 55.3) | 0.19 |
| Mental Quality of Life (median; IQR) | | 312 | 52.0 (42.7 – 55.7) | 151 | 53.8 (49.3 – 56.8) | <0.01 |
| ^a^ P-values of continues outcomes based on independent sample t-test or Mann-Whitney-U test. P-values of dichotomous and categorical outcomes are based on the Pearson Chi-Square test, the Fisher’s exact test or Fisher-Freeman-Halton exact test.  ^b^ Diagnosed by Rotterdam 2003 criteria (Fauser, 2004). | | | | | | |

| **Supplementary Table SII.** Anxiety and depression women with PCOS compared to ovulatory women | | | | |
| --- | --- | --- | --- | --- |
| **Anxiety and depression outcomes (HADS)** | **PCOS** ^a^  (*n* = 73) | **Ovulatory**  (*n* = 84) | **Mean difference ^b^** | **95% CI** ^c^ |
| Symptoms of anxiety (score; mean; SD) | 8.3 (3.9) | 8.1 (3.5) | 0.17 | -0.96 – 1.31 |
| Symptoms of depression (score; mean; SD) | 8.2 (3.7) | 7.5 (3.3) | 0.70 | -0.40 – 1.83 |
| HADS total score (score; mean; SD) | 16.5 (7.0) | 15.6 (6.2) | 0.87 | -1.18 – 3.01 |
| ^a^ Diagnosed by Rotterdam 2003 criteria (Fauser, 2004).  ^b^ The mean difference between the PCOS and ovulatory group was assessed with the independent sample t-test.  ^c^ Bias corrected and accelerated 95% CIs based on 5000 bootstrap re-samples; CI not containing zero indicate statistical significance. | | | | |

| **Supplementary Table SIII**. Quality of life in women with PCOS compared to ovulatory women | | | | | |
| --- | --- | --- | --- | --- | --- |
| **Quality of Life outcomes (SF-36)** | | **PCOS** ^a^  (*n* = 73) | **Ovulatory**  (*n* = 84) | **Mean difference** ^b^ | **95% CI** ^c^ |
| *Physical Component Summary (score; mean; SD)* | | 46.9 (8.5) | 46.4 (8.3) | 0.50 | -2.12 – 3.07 |
|  | Physical functioning, score (score; mean; SD) | 84.6 (19.1) | 85.1 (15.0) | -0.40 | -5.89 – 4.93 |
|  | Role limitations due to physical health (score; mean; SD) | 73.0 (37.0) | 80.7 (32.8) | -7.71 | -18.69 – 3.10 |
|  | Bodily pain (score; mean; SD) | 76.7 (24.0) | 74.5 (24.9) | 2.18 | -5.34 – 10.01 |
|  | General health (score; mean; SD) | 48.6 (4.3) | 48.5 (3.5) | 0.18 | -1.02 – 1.45 |
| *Mental Component Summary (score; mean; SD)* | | 47.8 (10.6) | 51.3 (9.2) | -3.43 | -6.64 – -0.39 |
|  | Social functioning domain (score; mean; SD) | 80.0 (21.4) | 85.1 (19.7) | -5.15 | -11.68 – 1.41 |
|  | Role limitations due to emotional problems (score; mean; SD) | 76.3 (38.7) | 89.3 (27.0) | -13.03 | -23.43 – -2.70 |
|  | Vitality domain (score; mean; SD) | 53.8 (20.7) | 58.8 (20.3) | -4.91 | -11.13 – 1.52 |
|  | Mental health/emotional well-being (score; mean; SD) | 72.3 (14.9) | 75.2 (15.9) | -2.86 | -7.63 – 1.98 |
| ^a^ Diagnosed by Rotterdam 2003 criteria (Fauser, 2004).  ^b^ The mean difference between the PCOS and ovulatory group was assessed with the independent sample t-test.  ^c^ Bias corrected and accelerated 95% CIs based on 5000 bootstrap re-samples; CI not containing zero indicate statistical significance. | | | | | |

| **Supplementary Table SIV**. Sexual function and intercourse frequency in women with PCOS compared to ovulatory women | | | | | | | | |
| --- | --- | --- | --- | --- | --- | --- | --- | --- |
|  | | | | | **Unadjusted** | | **Adjusted** ^d^ | |
| **Sexual function outcomes (MFSQ)** | **n** | **Ovulatory** | **n** | **PCOS** ^a^ | **Mean difference** ^b^ | **95% CI** ^c^ | **Mean difference** ^b^ | **95% CI** ^c^ |
| Sexual interest (score; mean; SD) | 84 | 25.6 (7.7) | 73 | 26.4 (6.6) | 0.79 | -1.50 – 3.05 | 0.06 | -2.40 – 2.52 |
| Sexual satisfaction (score; mean; SD) | 81 | 10.9 (2.8) | 70 | 11.2 (2.5) | 0.31 | -0.53 – 1.17 | 0.18 | -0.65 – 1.03 |
| Vaginal lubrication (score; mean; SD) | 67 | 15.5 (3.7) | 64 | 16.4 (3.0) | 0.91 | -0.23 – 2.02 | 1.03 | -0.21 – 2.29 |
| Orgasm, score (score; mean; SD) | 67 | 19.8 (5.6) | 64 | 20.4 (4.8) | 0.58 | -1.22 – 2.31 | 0.80 | -1.11 – 2.80 |
| Sex partner (score; mean; SD) | 79 | 18.8 (2.6) | 70 | 18.9 (2.1) | 0.04 | -0.71 – 0.83 | -0.14 | -1.01 – 0.73 |
| Total MFSQ (score; mean; SD) | 67 | 93.1 (14.7) | 64 | 94.4 (13.5) | 1.23 | -3.55 – 6.09 | 0.60 | -4.33 – 5.71 |
| Intercourse frequency (number per 4 weeks; mean; SD) | 67 | 5.5 (4.6) | 64 | 6.0 (5.8) | 0.48 | -1.26 – 2.36 | -0.28 | -2.10 – 1.80 |
| ^a^ Diagnosed by Rotterdam 2003 criteria (Fauser, 2004).  ^b^ The mean difference between the PCOS and ovulatory group was assessed with linear regression.  ^c^ Bias corrected and accelerated 95% CIs based on 5000 bootstrap re-samples; CI not containing zero indicate statistical significance.  ^d^ Adjusted for randomisation group within the initial RCT and attempting to conceive. | | | | | | | | |

| **Supplementary Table SV**. Questions in SF-36^a^ subscale ‘role limitations due to emotional problems’ | | | |
| --- | --- | --- | --- |
| During the **past 4 weeks** have you had any of the following problems with your work or other regular daily activities **as a result of any emotional problems** (such as feeling depressed or anxious)? | | | |
|  |  | Yes | No |
| 17. | Cut down the **amount of time** you spent on work or other activities |  |  |
| 18. | **Accomplishes less** than you would like |  |  |
| 19. | Didn’t do work or other activities as **careful** as usual |  |  |
| ^a^ SF-36: 36-Item Short Form Health Survey (SF-36) (Wendel-Vos et al., 2003). | | | |

**References in tables**

Fauser, B. C. J. M. (2004). Revised 2003 consensus on diagnostic criteria and long-term health risks related to polycystic ovary syndrome. *Fertility and Sterility*. https://doi.org/10.1016/j.fertnstert.2003.10.004

Wendel-Vos, G. C. W., Schuit, A. J., Saris, W. H. M., & Kromhout, D. (2003). Reproducibility and relative validity of the short questionnaire to assess health-enhancing physical activity. *Journal of Clinical Epidemiology*. https://doi.org/10.1016/S0895-4356(03)00220-8
